# Mutual interference is common and mostly intermediate in magnitude

**DOI:** 10.1186/1472-6785-11-1

**Published:** 2011-01-06

**Authors:** John P DeLong, David A Vasseur

**Affiliations:** 1 Department of Ecology and Evolutionary Biology, Yale University, New Haven, CT 06520 USA

## Abstract

**Background:**

Interference competition occurs when access to resources is negatively affected by the presence of other individuals. Within a species or population, this is known as mutual interference, and it is often modelled with a scaling exponent, *m*, on the number of predators. Originally, mutual interference was thought to vary along a continuum from prey dependence (no interference; *m *= 0) to ratio dependence (*m *= -1), but a debate in the 1990's and early 2000's focused on whether prey or ratio dependence was the better simplification. Some have argued more recently that mutual interference is likely to be mostly intermediate (that is, between prey and ratio dependence), but this possibility has not been evaluated empirically.

**Results:**

We gathered estimates of mutual interference from the literature, analyzed additional data, and created the largest compilation of unbiased estimates of mutual interference yet produced. In this data set, both the alternatives of prey dependence and ratio dependence were observed, but only one data set was consistent with prey dependence. There was a tendency toward ratio dependence reflected by a median *m *of -0.7 and a mean *m *of -0.8.

**Conclusions:**

Overall, the data support the hypothesis that interference is mostly intermediate in magnitude. The data also indicate that interference competition is common, at least in the systems studied to date. Significant questions remain regarding how different factors influence interference, and whether interference can be viewed as a characteristic of a particular population or whether it generally shifts from low to high levels as populations increase in density.

## Background

Competition has long been thought to be a major force shaping evolutionary and ecological processes [1]. Individuals compete for resources with other individuals, and this competition limits growth rate and population size [2]. Competition occurs in two major forms. Exploitation competition occurs when a resource is reduced in quantity because other individuals consume or control it. Interference competition occurs when access to a resource that is still present or available is reduced by interactions with other individuals. Such interactions may be aggressive or passive. When interference occurs among individuals of the same species or population, it is known as mutual interference [3,4].

Exploitation competition occurs because the rate of resource uptake (foraging) depends positively on resource availability. Models that describe how resource uptake is related to resource density are known as functional responses [5]. A linear (type I) functional response describing the per-capita rate of resource consumption of a consumer, *f*, can be written as:

(1) f(R,C)=aR

In this model, *R *is the resource density, *C *is the consumer density, and the attack efficiency, *a*, determines the proportion of the potential consumer-resource interactions that yield a consumption event. Equation 1 describes the foraging rate of a consumer based on mass action, because the total resource consumption by a population of consumers *C *is given by *aRC*. This functional response has been criticized because it describes a linear increase in consumption with resource density, whereas most consumers' consumption rates saturate at high resource density due to the time it takes to "handle" a unit of resource. This effect typically is captured by rescaling equation 1 by the time cost of handling prey, *h*, yielding a type II functional response [5]:

(2) $$f(R,C)=\frac{aR}{1+ahR}$$

Interference competition factors into the functional response in two ways. First, it alters the rate of interactions between the consumer and the resource. Thus, instead of a simple mass action term, the presence of other consumers reduces the rate of interactions in a decelerating manner that is well-described by including an interference parameter, *m*, as an exponent on the consumer number [6]:

(3) $$f(R,C)=\frac{aRC^{m}}{1+ahRC^{m}}$$

In equation 3, if *C *= 1 (or *m *= 0) the functional response reduces to equation 2. Most studies of mutual interference assume that this modification of mass action is the primary effect of interference, and, using this type of functional response model, attempt to quantify *m*^1 ^[6].

*^1 ^In this study, we adopt the notation of m without a minus (-) sign. In much of the previous work, m is given with a minus sign in models. We think this confuses matters, because the typically negative values of m help to see that it depresses foraging rates, which is obscured by focusing on m as a positive number*.

Interference also may have a secondary effect, which is to impose an additional cost that is time "wasted" interacting with other consumers [7,8]. In this analysis, we are concerned only with the value of *m *and the controversies surrounding its estimation and magnitude; we therefore do not consider the alternative models further.

The magnitude of *m *has been the subject of considerable debate. Early studies of interference typically found that *m *is between 0 and -1 [3,4]. Nonetheless, many studies continued to assume that *m *= 0, ignoring the effects of interference. In 1989, a study by Arditi and Ginzburg [9] suggested that what matters to a consumer is the ratio of resources to consumers and not just the absolute amount of resource. This argument gives rise to a functional response known as "ratio-dependent", with the ratio *R/C *replacing *R *[9]:

(4) $$f(R,C)=\frac{a\frac{R}{C}}{1+ah\frac{R}{C}}$$

Equation 4 is clearly just a special case of equation 3 with *m *= -1. The special case of *m *= 0 was thereafter named prey-dependent because in that case foraging rates depend only on the resource density and not the consumer density. This categorization marked the start of a new debate over the magnitude of *m*, with authors arguing for or against ratio- or prey-dependent functional responses [10-12]. In other words, authors argued that *m *was typically either -1 or 0, although some still suggested there was really a continuum between the two endpoints [9,11].

An upshot of the debate was that additional attention was given to how to properly estimate *m *from data. In the study of Arditi and Akçakaya [6], proponents of the ratio-dependent approach identified a bias in the original approach used by Hassell [4]. In short, the original approach is likely to underestimate the value of *m *because it fits a linear model to saturating data (see "Approaches to estimating mutual interference" for further details). Introducing and applying an unbiased method to data from previous studies, they found that most data produced estimates of *m *that were statistically indistinguishable from ratio dependence (*m *= -1) but significantly different from prey dependence (*m *= 0). Through time, additional researchers have formulated other approaches to estimating *m *[13], and more studies have been conducted using a variety of methods. Since then, several studies have suggested that intermediate mutual interference (i.e., somewhere between 0 and -1) is likely to be more common than either pure prey dependence (*m *= 0) or ratio dependence (*m *= -1) [14-16]; however, no effort has been made yet to synthesize the new and expanding literature on this topic.

In this study, we comprehensively review the literature on mutual interference and analyze the distribution of *m *values from all studies in which it was reported and from additional studies where it could be calculated from data shown in the original study. In this way, we address the question of whether interference is best described by the simplifications of ratio or prey dependence, or whether intermediate levels are most typical. We describe four different methods used to estimate *m*, but evaluate our hypothesis using only the two approaches viewed as unbiased. Nonetheless, we evaluate the estimates produced by potentially biased approaches to explore how they compare with the unbiased approaches. Finally, we suggest that a similar approach using metabolic rates rather than foraging rates may provide new insights into interference competition, and we evaluate several studies that used this approach as well.

## Approaches to estimating mutual interference

Below we describe the four methods used to estimate *m *in the literature and an additional method that holds potential for use in the future.

### Method 1 - attack efficiency assuming linear functional response

The original method for estimating *m *was described by Hassell and Varley [3]. The approach is to regress the log of the attack efficiency, *a*, (which, again, is the proportion of possible consumer-resource interactions that results in a consumption event) against the log of the consumer density, *C*, and take the slope of the relation as the estimate of *m*:

(5) log(a)=mlog(C)+α

The attack efficiency (sometimes referred to as the "area of discovery" [17]) is calculated using a linear functional response, typically written in a manner slightly different from equation 1 following [6]:

(6) $$R_{a}=R[1-\exp(-aCT)]$$

Here, *R *is the amount of resource provided in an experimental trial, *R*_a _is the total number of resource items consumed by all consumers, and *T *is the total time of the foraging experiment. If this functional response is used to estimate *a *at a variety of levels of *C*, then equation 5 can be used to estimate *m*. In this approach, the level of *R *does not matter because the functional response is assumed to be linear. However, this approach was criticized by Arditi and Akçakaya [6] because most functional response data are more consistent with a type II (saturating) model than a type I (linear) model. Thus, when fitting a straight line to data that are saturating, the estimate of *a *will be lower than it really is, and this will be particularly true as the number of consumers decreases toward one. The end result is that the relationship between *a *and *C *will be too shallow and *m *will be underestimated.

### Method 2 - attack efficiency assuming saturating functional response

This method corrects the bias of Method 1 and was developed by Arditi and Akçakaya [6]. Instead of using equation 6 to estimate *a*, a type II version is used, allowing simultaneous estimation of both *a *and the handling time, *h*:

(7) $$R_{a}=R[1-\exp(-aCT+ahR_{a})]$$

Then, with estimates of *a *for a variety of levels of *C*, one again uses equation 5 to estimate *m*. This approach prevents *a *from being suppressed as a result of fitting a line to a curve.

### Method 3 - fitting data with variation in *R *and *C *to a functional response

This method takes data on resource uptake rates in relation to both resource and consumer density and fits the functional response to all the data at once. In this approach one dispenses with the need to first calculate *a *for a variety of levels of *C *and then regress log(*a*) against log(*C*). With this approach the parameters *a*, *m*, and *h *are all produced in a single fitting procedure.

### Method 4 - fitting foraging rate data with variation in *C *to a functional response

This method makes use of data sets where variation in consumer number and not variation in resource level is available. In Method 4, instead of using *a *as the dependent variable, one regresses the log of the per-capita kill, oviposition, or foraging rate against the log of the consumer number:

(8) $$\log(\frac{R_{a}}{C})=m\log(C)+\alpha$$

This produces a similar range of values as the other methods, but these estimates are likely to be underestimates. Rearranging equation 3, we see that fitting equation 8 forces an increase in the intercept as the consumer level increases, because the denominator of the term within the parenthesis gets smaller as *C *increases:

(9) $$f(R,C)=\left(\frac{aR}{1+ahRC^{m}}\right)C^{m}$$

To correct this, one could simply fit equation 3 directly to the set of data, even with no knowledge of the level of *R*, with the understanding that *aR *is produced as a combined parameter rather than *a*.

### Method 5 - fitting metabolic rate data with variation in *C *to a functional response

We hypothesize that Method 4 could be extended to utilize metabolic rate as a dependent variable rather than kill or resource uptake rates. The metabolic rate of an organism is the total sum of all energetic transformations occurring in its body and depends on the supply of substrates, most of which come in the form of food as described by the functional response. For some organisms, the time scales of resource uptake and utilization are quite small and metabolic rate will rapidly track the foraging rate, causing metabolic rate to respond to consumer density in the same way as resource uptake rate. Metabolic rate may be measured as oxygen consumption or heat production.

## Results

**Figure 1:**
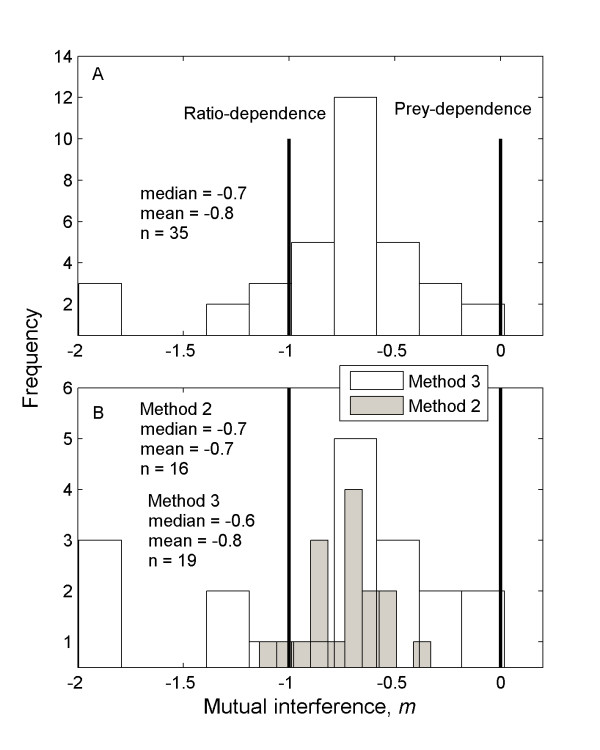
**The distribution of unbiased mutual interference values in the literature**. **A**. The distribution of *m *estimates gathered from studies in the literature that used the unbiased approaches of Methods 2 and 3 (see "Approaches to estimating mutual interference" for details). This histogram shows that most estimates of mutual interference cover continuously the range from a little above 0 to a little below -1. Two estimates were particularly large, at -2, but were highly precise and thus cannot be dismissed as errors. The special cases of prey dependence, when *m *= 0, and ratio dependence, when *m *= -1, are shown, and although both occur, intermediate interference is the most common state. **B**. A comparison of the distribution for the two unbiased approaches. Both approaches overall produce similar histograms, but the large values of -2 were produced using Method 3.

**Figure 2:**
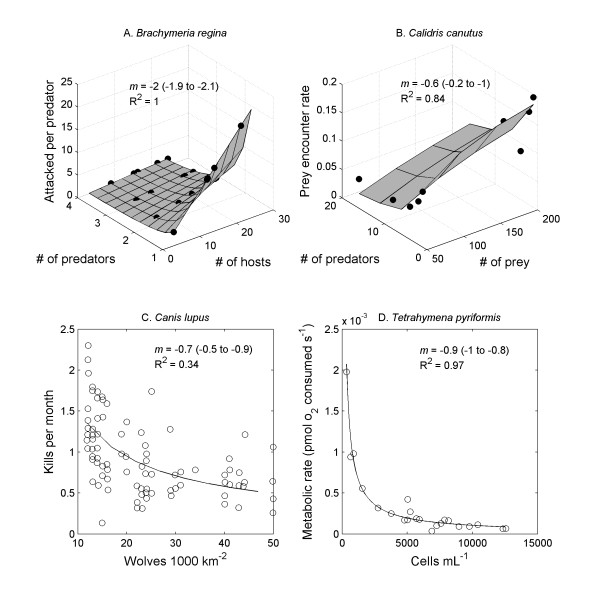
**Examples of the effect of consumer and resource density on resource uptake rate**. **A**. In some studies, a range of consumer and resource densities was available, allowing the use of Method 3 to fit all data to a functional response. In this case, the parasitoid *Brachymeria regina *parasitized the butterfly *Pieris rapae*. Data from [18]. **B**. In other studies, a broader range of resource densities than consumer densities were available. This study shows the success of knots (*Calidris canutus*) foraging on mussels (*Mytilus edulis*). Data from [20]. **C**. In several studies, only variation in consumer density could be related to resource uptake rates, in which case Method 4 was used. These data show the kill rate for wolves (*Canis lupus*) foraging on moose (*Alces alces*). Data from [16]. **D**. In an extension of Method 4, some studies reported variation in metabolic rates associated with population density. In this case, the protist *Tetrahymena pyriformis *growing in axenic culture. Data from [29].

We included 51 estimates of *m *from 37 studies in our review. Of the 51 estimates, 35 (69%) were produced using the unbiased approaches of Methods 2 and 3 (see Methods and Additional file 1 - data set). The data suggest that it is most often the case that consumers have interference interactions within their populations that are intermediate in magnitude. The unbiased estimates were statistically indistinguishable from prey dependence in only one instance but indistinguishable from ratio dependence in nine. Combining the two unbiased approaches, *m *values ranged from -2 to 0, with a median of -0.7, a mean of -0.8, and a mode of -0.7 (Figure 1A). Not including the three values of -2, the range is -1.3 to 0, with a median of -0.7. The unbiased methods 2 and 3 generally produced similar estimates (Figure 1B), but Method 3 produced some particularly large estimates of *m*, including some that were approximately -2, far outside the previously expected range of values (Figure 1B). It is worth noting that two of these estimates of -2 came from the same study [18], and that the fits for these data were exceptionally good (*R*^2 ^= 1) with very narrow confidence intervals for the estimates (Figure 2A, Additional file 1 - data set). The other high estimate came from one of the only studies to date conducted in a natural setting, for wolves predating moose on Isle Royale [19].

**Figure 3:**
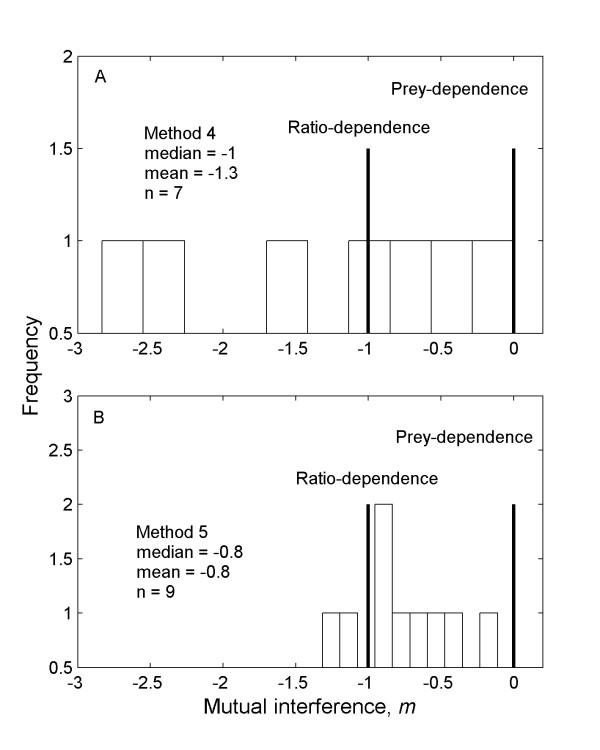
**The distribution of mutual interference values in the literature from potentially biased methods**. **A**. The distribution of *m *estimates gathered from studies in the literature that used the potentially biased approach of Method 4 (see " Approaches to estimating mutual interference" for details). This histogram shows that most estimates of mutual interference using this approach are similar to the unbiased approaches, but there are several large estimates that were produced with very low confidence (see Additional file 1 - data set). The special cases of prey dependence, when *m *= 0, and ratio dependence, when *m *= -1 are shown. **B**. The distribution of *m *estimates using the new approach of Method 5. This histogram shows that most estimates of mutual interference using this approach are similar to the unbiased approaches, but fitting of the whole functional response model often failed, and thus most of these are produced using the reduced power-function alternative.

The potentially biased Method 4 produced estimates that were mostly in the same range as Methods 2 and 3 (Figure 3A). Method 4 also produced some estimates of *m *that were of much larger magnitude (~-2.3 to -2.8), but in these cases the fits were not good and the confidence intervals for the estimates were very large (e.g., -4 to -0.6), suggesting that there was insufficient data. Fitting instead the simplified power function with fewer parameters to estimate (equation 8) produced narrower confidence intervals and lower estimates of *m *(Additional file 1 - data set). In the one available comparison, Method 4 produced a smaller estimate of *m *than Method 3 for "mixed scale" data on wolves predating moose on Isle Royale [16,19] (Figure 2C).

Method 5, in which we extended the use of fitting a type II functional response to metabolic rates, produced a range of estimates similar to the overall unbiased approaches (Figure 3B). These estimates therefore generally support the idea that interference effects may be observable in both the foraging behaviour and the energetic fluxes of organisms. This approach with metabolic rates generally did not work well with overall fitting of equation 3, as the fitting procedure often failed to converge. As with Method 4, however, applying the simplified power function (equation 8) produced strong fits (Additional file 1 - data set). Some examples of data fitted using methods 3 - 5 are shown in Figure 2.

## Discussion

Our analysis indicates that mutual interference can be characterized as highly variable with a tendency toward intermediate levels around -0.6 to -0.7 (Figure 1A). This result is consistent with the recent suggestion that along the continuum from ratio to prey dependence, intermediate interference will be the most commonly observed level [14-16]. Our study builds on previous reviews in more than doubling the number of unbiased estimates of *m *analyzed [6,13]. In addition, our study helps to resolve the long-standing debate over whether prey or ratio dependence is the better simplification. Our large sample shows that most studies give an interference value that is intermediate on the interval formed by the ratio- and prey-dependent special cases. The data provide very little support for prey dependence but some support for ratio dependence.

Despite this advance, there remain important unanswered questions about interference. In particular, what are the factors that generate a particular level of interference? Interference is generated by interactions such as passive or aggressive physical contact, but it may be altered by behaviours that reduce contact such as spacing and territoriality, social interactions that increase per-capita resource uptake rates, or prey switching [20]. These general factors may be associated with body size, prey type, movement rates and patterns, search strategies, temperature, habitat type, and many others possible traits [14]. Very few of these factors have been evaluated for their effect on interference, but this is clearly an important future direction. For example, in one study (which was not included in the histogram because it used the biased Method 1), the estimate of *m *for female *Trioxys indicus *(a parasitoid) was the same among three different foraging environments both with and without male interference [21]. The intercept of the relationship between attack efficiency and female density varied, suggesting that the environment may alter foraging rates independently of how it affects interference interactions. Also, in a study on wolves predating moose, different levels of interference were observed depending on the scale of observation (interference within packs versus among the whole population) [19], suggesting a strong role for the frequency of interaction and spatial context on setting interference levels.

The median and mean values of *m *are intermediate, but there is little theoretical explanation for why this value and not some other value would be most common. Indeed, the existence of values in the range of -2 suggests that our preconceptions of the range and typical nature of interference is not as good as previously thought. However, one previous study using indirect methods found even more severe interference levels [22], and the pack-scale level of interference in wolves was reported as -1.85 [19]. The -2 values cannot be dismissed as outliers because they are among the most precise estimates in the entire data set (meaning that they have very tight confidence intervals) and were generated using unbiased methods. The wolf estimate had larger confidence intervals but was derived from a natural setting, lending it greater weight than the laboratory studies. The historical focus of -1 or 0, along with most previous estimates of *m*, may have made it difficult to conceive of values as severe as -2. Yet, -2 would be expected given mass action acting on the consumers themselves. Just as the rate of interactions between a consumer and its resource is given by their product, the rate of interaction between consumers may be given as the consumers squared, which would lead to a reduction in consumer-resource interactions described by *C*^-2^. Assessing this possibility will require much closer scrutiny to the mechanisms - particularly the rate of contact among consumers - when studying interference.

Another open question is whether interference is characteristic of a population at a given time and place or whether interference levels may vary within a given time and place as the density of consumers changes. We will refer to these two scenarios as "characteristic" and "shifting", respectively. Characteristic interference is the implicit scenario of most studies that have measured interference, where interference is simply estimated from data and used to understand some aspect of the population's behaviour. Given that *m *is a parameter in equation 3, the assumption is that this level of interference applies to all levels of population size.

Alternatively, interference could shift from low to high levels as population size increases. This is the view taken originally by Hassell and Varley [3] and more recently by Ginzburg and Jensen [15]. Ginzburg and Jensen argue that at some low level of consumer density, interactions should be rare and mutual interference should come into play only as a population grows above some threshold level. Their spatial depiction of this process shows that as consumers become denser, the home ranges they use to acquire resources overlap more with those of other consumers, generating more interference. Similarly, Tyutyunov et al. [23] suggested that mutual interference may grade continuously from 0 to -1 as the population grows, and they derive a continuous-form functional response to describe this change.

At a low enough density of competitors, individuals may rarely encounter each other, so it does seem likely that there would be a minimum density for mutual interference to engage. Both of the switching alternatives (discrete or continuous shift from 0 to -1) imply that log-log plots of attack efficiency or resource uptake versus consumer number would show non-linearities, either a discrete bend from 0 to -1 in the former or a gradual curving in the latter. There is some evidence for such shifting in the analysis of Arditi and Akçakaya [6]. In their Figure [3], a levelling of attack efficiency at low densities is apparent in their data sets 9, 10, and 15, but this levelling is not observed in most data sets. Also, it is unclear why interference must increase to -1 at higher consumer densities. At carrying capacity, the increase in resource uptake by the population must be insufficient to generate additional individuals. This need not be a place where *m *= -1, but just some density where the drop in per-capita resources leads to equal birth and death rates. Thus, even if mutual interference engages at a particular low density, above that density, the observed value of *m *still may be a reflection of the types of interactions characterizing the population.

Regardless of its value, mutual interference may have significant ramifications for ecological processes. For example, numerous theoretical studies have shown that the presence of mutual interference alters the stability and numerical properties of populations and food webs (see [24] and references therein). Similarly, given that interference competition should be additive to exploitative competition, a particular value of *m *could have implications for inter-specific interactions as well, in particular, competitive outcomes [25,26]. Overall, our results indicate that interference is usually present, at least in the studies conducted to date, suggesting that interference effects shown in theoretical studies may be important.

## Conclusions

An analysis of unbiased estimates of *m *indicates that interference is highly variable but tends toward intermediate levels. Research on interference should move past prior disagreements over ratio and prey dependence and focus on understanding the factors that produce interference and determine whether interference is characteristic or shifting.

## Methods

We intensively searched the literature, with no taxonomic restrictions, for studies that reported values of *m *or that we could use to calculate values of *m*. We include information about each of these studies, the methods used, and the estimates of *m *in Additional file 1 - data set. Because of potential bias, we do not include any studies in our results or appendix that estimated *m *using Method 1 (all methods described in "Approaches to estimating mutual interference" above) unless the data were re-analyzed by Method 2 or 3 in another study. Arditi and Akçakaya [6] used Method 2 to reanalyze data from 15 studies, and we included these estimates in our compilation along with an estimate from one additional study that used this approach. We included one data point from Skalski and Gilliam [13]. Although Skalski and Gilliam [13] analyzed data from 19 studies, they only reported *m *values for five studies (for the other data sets they reported parameters for the alternative functional response [7,8]), three of which also had been analyzed by Arditi and Akçakaya [6]. Skalski and Gilliam did not include all the data available in [27], who also used Method 3, so we included all three original estimates of *m *from [27] instead. We analyzed nine additional data sets extracted from the literature using Method 3. We fit equation 3 to these data using ordinary least squares regression in the surface fitting tool in Matlab^©^. We used Methods 4 and 5 to estimate *m *for 7 and 9 data sets, respectively, extracted from the literature, again using ordinary least squares regression. These results are presented separately from the unbiased results of Methods 2 and 3, both for comparative purposes and for completeness. We did not include indirect estimates of *m *derived from fitting models to time-series or abundance data (e.g., [22,28]).

## Authors' contributions

JPD conceived of the research, carried out the literature search and data analysis, and drafted the manuscript. DAV assisted with study design, interpretation, and writing of the manuscript. Both authors read and approved the final manuscript.

## Supplementary Material

Additional file 1 **Dataset of mutual interference**. This pdf file contains the values of mutual interference used in our analysis. These are estimates found in the literature, plus our newly calculated values, with species, the methods used, and the data sources. Click here for file
