# Supplementary material for: Mutual interference is common and mostly intermediate in magnitude
Source: BMC Ecol. 2011 Jan 6;11:1. doi: 10.1186/1472-6785-11-1 (PMC3024213; doi:10.1186/1472-6785-11-1)
Supplement: Additional file 1 — Dataset of mutual interference. This pdf file contains the values of mutual interference used in our analysis. These are estimates found in the literature, plus our newly calculated values, with species, the methods used, and the data sources. [file 1472-6785-11-1-S1.PDF]

Addition file for: DeLong JP, Vasseur DA: **Mutual interference is common and mostly intermediate in magnitude**. *BMC Ecology* 2011

This file contains data used to assess the distribution of mutual interference values, *m*, reported in the literature. It includes information on the species and taxonomic affiliation of the consumer; the method of estimation (see Methods for full descriptions); range of estimate includes as appropriate the 95% confidence intervals (CI), standard error (SE), or the range of single estimates reported; and the sources for the data.

Consumer species	Taxa[1]	Meth	*m*	Error	R2	Notes for Method 4 and 5	*m* calculated in	Original data source
Daphnia pulex	C	2	-1.05	± 0.36 (SE)			[1]	[2]
Clupea harangus	F	2	-0.54	± 0.16 (SE)			[1]	[3]
Clupea harangus	F	2	-0.73	± 0.17 (SE)			[1]	[3]
Clupea harangus	F	2	-0.66	± 0.17 (SE)			[1]	[3]
Amblyseius degenerans	I	2	-0.50	± 0.09 (SE)			[1]	[4]
Nasonia vitripennis	I	2	-1.14	± 0.15 (SE)			[1]	[5]
Tribolium castaneum	I	2	-0.83	± 0.09 (SE)			[1]	[6]
Trichogramma evanescens	I	2	-0.89	± 0.07 (SE)			[1]	[5]
Trichogramma pretiosum	I	2	-0.33	± 0.14 (SE)			[1]	[7]
Trioxys indicus	I	2	-0.64	± 0.15 (SE)			[1]	[8]
Trioxys indicus	I	2	-0.62	± 0.21 (SE)			[1]	[8]
Trioxys indicus	I	2	-0.66	± 0.15 (SE)			[1]	[8]
Trybliographa rapae	I	2	-0.70	± 0.22 (SE)			[1]	[9]
Urosalphinx cinerea	M	2	-0.87	± 0.35 (SE)			[1]	[10]
Phytoseiulus persimilis	I	2	-0.92	± 0.16 (SE)			[1]	[4]
Aphidius matricariae	I	2	-0.76	-			[11]	[11]
Back swimmer	I	3	-0.33	-0.20 to -0.43 (CI)			[12]	[13]
Bracon hebetor	I	3	-0.63	-0.59 and -0.66 (2 estimates)			[14]	[14]
Bracon hebetor	I	3	-0.45	-0.41 and -0.49 (2 estimates)			[14]	[14]
Stenostomum virginianum	W	3	-0.67	± 0.11 (SE)			[15]	[15]
Carcinus maenas	C	3	-0.90	-			[16]	[16]
Hemigrapsus sanguineus	C	3	-0.10	-			[16]	[16]
Polistes dominulus	I	3	-0.50	-0.40 to -0.61 (CI)			[17]	[17]
Polistes dominulus	I	3	-0.32	-0.26 to -0.38 (CI)			[18]	[18]
Thanasimus dubius	I	3	-1.00	-0.58 to -1.43 (CI)			[19]	[19]
Arenaria interpres	B	3	-0.63	-0.22 to -1.04 (CI)	0.84		This study	Figure 4 from [20]
Calidris canutus	B	3	-0.55	-0.43 to -0.66 (CI)	0.99		This study	Figure 4 from [20]

Tetragoneuria cynosure larvae	I	3	-1.27	-1.04 to -1.50 (CI)	0.98		This study	Figure 2 from [21]
Apanteles glomeratus	I	3	-0.63	-0.28 to -0.97 (CI)	0.88		This study	Table 1 from [22]
Brachymeria regina	I	3	-1.99	-1.98 to -2.06 (CI)	1.00		This study	Table 1 from [22]
Pteromalus puparum	I	3	-1.99	-1.99 to -2.00 (CI)	1.00		This study	Table 1 from [22]
Anagyrus sp.	I	3	0.02	-0.50 to 0.54 (CI)	0.86		This study	Figure 3a from [23]
Trichogramma minutum	I	3	-1.25	-1.48 to -1.02 (CI)	0.72		This study	Figure 1a,b from [24]
Callinectes sapidus	C	3	-0.65	-1.17 to -0.12 (CI)	1.00		This study	Figure 1 from [25]
Canis lupus "pack" scale	M	3	-1.85	-2.12 to -1.53			[26]	[26]
Canis lupus	M	4	-0.70	-0.47 to -0.94 (CI)	0.34	Identical when $h = 0$	This study	Figure 1 from [27]
Calidris canutus	B	4	-2.83	-7.54 to 1.88 (CI)	0.95	With $h = 0$, $m = -0.47$ (-0.9661, 0.02911)	This study	Figure 3 from [28]
Cheiropachus guadrum	I	4	-1.062	-1.53 to -0.59	0.73	Identical when $h = 0$	This study	Figure 4 from [29]
Anagrus delicatus	I	4	-0.422	-2.84 to 2.00 (CI)	0.18	With $h = 0$, $m = -0.30$ (-0.50, 0.10)	This study	Figure 3b from [30]
Anagrus delicatus	I	4	-1.599	-4.06 to 0.86 (CI)	0.51	With $h = 0$, $m = -0.69$ (-1.14, -2.3)	This study	Figure 7b from [31]
Woodruffia metabolica	P	4	0.00	-0.16 to 0.15	0.00	Full fit failed, power function used	This study	Figure 7 from [32]
Didinium nasutum	P	4	-2.30	-3.99 to -0.60	0.34	With $h = 0$, $m = -0.24$ (-0.35, -0.13)	This study	Figure 4 from [33]
Amoeba proteus	P	5	-0.55	-1.45 to 0.36	0.67	With $h = 0$, $m = -0.54$ (-0.66, -0.42)	This study	Figure 4 from [34]
Tetrahymena pyriformis	P	5	-0.11	-0.09 to -0.13	0.96	Full fit failed, power function used	This study	Figure 2 from [35]
Tetrahymena pyriformis	P	5	-0.89	-0.98 to -0.80	0.97	Full fit failed, power function used	This study	Figure 2 from [36]
Tetrahymena pyriformis	P	5	-0.92	-0.83 to -1.01		Full fit failed, power function used	This study	Figure 2 from [37]
Blepharisma sp.	P	5	-1.32	-5.37 to 2.73	0.90	With $h = 0$, $m = -0.81$ (-1.35, -0.28)	This study	Figure 2 from [37]
Chlamydomonas reinhardtii	A	5	-0.76	-0.17 to -1.35		Full fit failed, power function used	This study	Figure 2 from [37]
Galdieria sulfuraria	A	5	-0.66	-0.26 to -1.07		Full fit failed, power function used	This study	Figure 2 from [37]
Calanus finmarchicus	C	5	-0.40	-0.19 to -0.60	0.84	Identical when $h = 0$	This study	Table 2 from [38]
Simocephalus vetulus	C	5	-1.12	-1.35 to -0.90		Full fit failed, power function used	This study	Table 3 from [39]

[1] Taxa abbreviations: A = algae, B = bird, C = crustacean, W = flatworm, I = insect, L = mollusk, M = mammal, P = protist, S = snail

References

1. Arditi R, Akçakaya HR: **Underestimation of mutual interference of predators**. *Oecologia* 1990, **83**:358-361.

2. Chant D, Turnbull A: **Effects of predator and prey densities on interactions between goldfish and Daphnia pulex (de Geer).** *Can. J. Zool* 1966, **44**:285-289.

3. von Westernhagen H, Rosenthal H: **Predator-prey relationship between Pacific herring, Clupea harengus pallasi, larvae and a predatory hyperiid amphipod, Hyperoche medusarum.** *Fish Bull* 1976, **74**:669-674.

4. Eveleigh E, Chant D: **Experimental studies on acarine predator–prey interactions: the effects of predator density on prey consumption, predator searching efficiency, and the functional response to prey density (Acarina: Phytoseiidae)**. *Can. J. Zool* 1982, **60**:611-629.

5. Edwards R: **The area of discovery of two insect parasites, Nasonia vitripennis (Walker) and Trichogramma evanescens Westwood, in an artificial environment**. *Canadian Entomologist* 1961, **93**:475-481.

6. Mertz DB, Davies RB: **Cannibalism of the pupal stage by adult flour beetles: an experiment and a stochastic model**. *Biometrics* 1968, **24**:247-275.

7. Kfir R: **Functional response to host density by the egg parasite Trichogramma pretiosum**. *BioControl* 1983, **28**:345-353.

8. Kumar A, Tripathi C: **Parasitoid–host relationship between Trioxys (Binodoxys) indicus Subba Rao &Sharma (Hymenoptera: Aphidiidae) and Aphis craccivora Koch (Hemiptera: Aphididae): effect of host plants on the area of discovery of the parasitoid**. *Can. J. Zool* 1985, **63**:192-195.

9. Jones TH, Hassell MP: **Patterns of parasitism by <i>Trybliographa rapae</i>, a cynipid parasitoid of the cabbage root fly, under laboratory and field conditions**. *Ecological Entomology* 1988, **13**:309-317.

10. Katz CH: **A nonequilibrium marine predator-prey interaction**. *Ecology* 1985, **66**:1426-1438.

11. Tahriri S, Talebi AA, Fathipour Y, Zamani AA: **Host stage preference, functional response and mutual interference of <i>Aphidius**

matricariae</i> (Hym.: Braconidae: Aphidiinae) on <i>Aphis fabae</i> (Hom.: Aphididae). *Entomological Science* 2007, **10**:323-331.

12. Skalski GT, Gilliam JF: **Functional responses with predator interference: viable alternatives to the Holling type II model**. *Ecology* 2001, **82**:3083-3092.

13. Uttley M: **A laboratory study of mutual interference between freshwater invertebrate predators.** 1980.

14. Taylor AD: **Host effects on functional and ovipositional responses of Bracon hebetor**. *Journal of Animal Ecology* 1988, **57**:173-184.

15. Kratina P, Vos M, Bateman A, Anholt B: **Functional responses modified by predator density**. *Oecologia* 2009, **159**:425-433.

16. Griffen BD, Delaney DG: **Species invasion shifts the importance of predator dependence**. *Ecology* 2007, **88**:3012-3021.

17. Schenk D, Bersier L, Bacher S: **An experimental test of the nature of predation: neither prey- nor ratio-dependent**. *Journal of Animal Ecology* 2005, **74**:86-91.

18. Tschanz B, Bersier L, Bacher S: **Functional responses: a question of alternative prey and predator density**. *Ecology* 2007, **88**:1300-1308.

19. Reeve JD: **Predation and bark beetle dynamics**. *Oecologia* 1997, **112**:48-54.

20. Vahl WK, van der Meer J, Weissing FJ, van Dullemen D, Piersma T: **The mechanisms of interference competition: two experiments on foraging waders**. *Behav. Ecol.* 2005, **16**:845-855.

21. Crowley PH, Martin EK: **Functional responses and interference within and between year classes of a dragonfly population**. *Journal of the North American Benthological Society* 1989, **8**:211-221.

22. Hassan S: **The area of discovery of Apanteles glomeratus (Hymenoptera: Braconidae), Pteromalus puparum (Pteromalidae) and Brachymeria regina (Chalcididae)**. *Entomologia Experimentalis et Applicata* 1976, **20**:199-205.

23. Chong J, Oetting RD: **Functional response and progeny production of the Madeira mealybug parasitoid, Anagyrus sp. nov. nr. sinope: The effects of host and parasitoid densities**. *Biological Control* 2006, **39**:320-328.

24. Mills NJ, Lacan I: **Ratio dependence in the functional response of insect parasitoids: evidence from <i>Trichogramma minutum</i>**

foraging for eggs in small host patches. *Ecological Entomology* 2004, **29**:208-216.

25. Mansour R, Lipcius R: **Density-dependent foraging and mutual interference in blue crabs preying upon infaunal clams.** *Mar. Ecol. Prog. Ser.* 1991, **72**:239-246.

26. Jost C, Devulder G, Vucetich JA, Peterson RO, Arditi R: **The wolves of Isle Royale display scale-invariant satiation and ratio-dependent predation on moose**. *Journal of Animal Ecology* 2005, **74**:809-816.

27. Vucetich JA, Peterson RO, Schaefer CL: **The effect of prey and predator densities on wolf predation**. *Ecology* 2002, **83**:3003-3013.

28. van Gils JA, Piersma T: **Digestively constrained predators evade the cost of interference competition**. *J Anim Ecol* 2004, **73**:386-398.

29. Lozano C, Kidd N, Jervis M, Campos M: **Effects of parasitoid spatial heterogeneity, sex ratio and mutual interference on the interaction between the olive bark beetle <i>Phloeotribus scarahaeoides</i> (Col., Scolytidae) and the pteromalid parasitoid <i>Cheiropachus quadrum</i> (Hym., Pteromalidae)**. *Journal of Applied Entomology* 1997, **121**:521-528.

30. Cronin JT, Strong DR: **Superparasitism and mutual interference in the egg parasitoid <i>Anagrus delicatus</i> (Hymenoptera: Mymaridae)**. *Ecological Entomology* 1993, **18**:293-302.

31. Cronin JT, Strong DR: **Substantially submaximal oviposition rates by a mymarid egg parasitoid in the laboratory and field**. *Ecology* 1993, **74**:1813-1825.

32. Salt GW: **Predation in an experimental protozoan population (Woodruffia-Paramecium)**. *Ecological Monographs* 1967, **37**:113-144.

33. Salt GW: **Predator and prey densities as controls of the rate of capture by the predator Didinium nasutum**. *Ecology* 1974, **55**:434-439.

34. Nässberger L, Monti M: **Assessment of overall metabolism in Amoeba proteus measured by a microcalorimetric method**. *Protoplasma* 1984, **123**:135-139.

35. Finlay B, Span A, Ochsenbein-Gattlen C: **Influence of physiological state on indices of respiration rate in protozoa.** *Comparative Biochemistry and Physiology* 1983, **74**:211-219.

36. DeLong JP, Hanson DT: **Metabolic rate links density to demography in Tetrahymena pyriformis.** *The ISME Journal* 2009, **2009**:1:9.

37. DeLong JP, Hanson DT: **Density-dependent individual and population-level metabolic rates in a suite of single-celled eukaryotes.** *The Open Biology Journal* 2009, **2**:32-37.

38. Zeiss FR: **Effects of population densities on zooplankton respiration rates**. *Limnol Oceanog* 1963, **8**:110-115.

39. Hoshi T: **Studies on physiology and ecology of plankton XII. Changes in O2-consumption of the daphnid, Simocephalus vetulus, with the decrease of O2-concentration.** *Sci Rep Tokohu Univ Biol* 1957, **23**:27-33.
